# Perspectives of community-dwelling older adults with dementia and their carers regarding their oral health practices and care: rapid review

**DOI:** 10.1038/s41405-021-00091-4

**Published:** 2021-11-22

**Authors:** S. KC, M. Aulakh, S. Curtis, S. Scambler, J. E. Gallagher

**Affiliations:** Faculty of Dentistry, Oral & Craniofacial Sciences, King’s College London, Denmark Hill Campus, Bessemer Road, London, SE5 9RS UK

**Keywords:** Dentistry, Gerodontics

## Abstract

**Aim:**

To review evidence on oral health practices, beliefs/views and experiences of community-dwelling older adults living with dementia, including their carers.

**Materials and methods:**

A search of key terms across six databases including Pubmed, Web of Science and OVID (Embase, MEDLINE [R] and PsycINFO) and Google Scholar was conducted, supplemented by reference screening. The Mixed Methods Appraisal Tool (MMAT) 2018 was used to assess the methodological quality.

**Results:**

Eighteen studies reported across 19 papers were included in the review. Papers largely focused on normative needs (*n* = 13), whilst also reporting oral health-related experiences (*n* = 2), practices (*n* = 7), and beliefs/views (*n* = 9), of community dwellers with dementia. Generally, people living with dementia presented with poor oral and dental health, the exception being one study where dental care was integrated with memory clinic services. Maintenance of oral health focused only on toothbrushing. Overall, people living with dementia have reduced capacity for self-performed oral hygiene and high reliance on caregivers. There was a paucity of evidence on their perceptions of oral health and quality of life, the findings of which were equivocal, with weak evidence suggesting possible difficulty in identifying and communicating their needs. Experiences of accessing dental care, when explored, appear to be system dependent.

**Conclusion:**

There was limited research evidence on oral health-related practices, beliefs/views and experiences of people with dementia. Recommendations for future research are presented.

## Introduction

Dementia is one of the biggest health and social care challenges facing the world today. Globally, an estimated 50 million people are currently diagnosed with dementia, with a predicted threefold increase by the year 2050.^1^

As a neurodegenerative disorder, dementia is characterised by a chronic decline in cognitive and motor functions, which increases in risk with age.^2^ The United Kingdom (UK) reports a high level of disease, with the latest figures suggesting an estimated 850,000 people living with a diagnosis of dementia.^3^ With the population share of later-life age groups above the age of 65 years predicted to increase to 19.8 million over the next 50 years,^4^ the age-related disease burden is on the rise, including the challenges presented by dementia. The prevalence is predicted to reach over two million people by the year 2051, which will significantly add to the existing economic burden of dementia which is estimated at £26.3 billion per annum to the national economy.^3^

A concurrent shift in the oral health profile of older adults means that people are also retaining more of their natural teeth. The last published Adult Dental Health Survey within the UK, conducted in 2009, reported that 53% of adults over the age of 85 years have retained an average of 14 natural teeth.^5^ Also, the volume of ‘edentate’ adults had fallen to 6%, which represents an all-time recorded low.^6^ Therefore, the link between dementia and oral health is of significant concern in an ageing society, as good oral health is an essential component of active ageing, social participation, communication and general wellbeing.^7^

Research, albeit limited in volume, suggests that dementia is associated with worse oral health, although a number of recent systematic reviews highlight that the nature and direction of association is still unclear.^8,9^ There is greater awareness of the oral health challenges related to dementia, including tooth loss, periodontal disease risk, caries risk and increased prevalence of orofacial pain.^10^ Furthermore, increasing evidence also highlights a relationship between oral health conditions, wider negative health and frailty in people diagnosed with dementia, associated with systemic inflammatory responses, medication use, dietary changes and malnutrition.^11^ However, similar to people with other neurodegenerative disorders or disability, people with dementia are at a reduced capacity to maintain their oral health. The loss of cognitive and motor functions restricts their ability to take care of their oral and general health.^12^ Also, access to regular dental services and professional care is increasingly challenged, due to a rapid decline in health as dementia progresses.^13^ Issues unique to dementia such as, communication barriers, resistance, and behavioural difficulties further restrict the type of care that can be delivered.^14^ Therefore, people living with dementia are at a risk of poorer oral health and they present high treatment needs, yet timely care access and type of care they receive is more restricted than the general population.

While the evidence on dementia-related oral health challenges is growing, studies so far have mostly been conducted in residential facilities, despite the fact that most people with dementia live in the community.^15^ Older people who are no longer able to live independently are often admitted in to formal care facilities such as nursing homes, at which stage, they frequently present with poorer oral health and extensive dental care needs.^16^ However, limited information is available about the unique experiences of those people living in the community with a dementia diagnosis (including their carers) to understand how they view their health and what they do to manage their oral and dental health. Moreover, considering that oral disease is largely preventable and oral health can be maintained through self/home care management and routine clinical care, the lack of community-level focus presents missed opportunities to improve the lives of people with dementia. An understanding of the perspectives of community dwellers with dementia is, critical to support timely, person-centred care that is most helpful to them in their dementia journey. It is equally important to acknowledge the views and voices of the carers alongside people with dementia support a shared decision-making process.^17^ As research on the perspectives of community dwellers with dementia regarding their oral health is emerging, a synthesis and review of evidence are thus, required. Therefore, this rapid review was conducted to answer the following key questions:

1. What do older adults, diagnosed with dementia (and their carers) do to look after their oral health?
2. What are their beliefs and views about oral health/oral health care?
3. What are their oral health-related experiences?
4. What changes occur over time in their dementia journey?

The aim of this review is, therefore, to review existing evidence on oral health practices, beliefs/views and experiences of community-dwelling older adults living with dementia including their carers.

## Materials and methods

### Protocol and registration

This study has been conducted systematically according to Khangura’s methodology^18^ and reported according to the Preferred Reporting Items for Systematic Reviews and Meta-Analyses (PRISMA).^19^ Rapid reviews follow the process of systematic reviews with methodological adjustments for an accelerated assessment of the latest evidence available.^20^ Our review included a number of verified adaptations to a systematic review approach (including searching key databases, study screening by paired two groups, data extraction as a dyad and completion of review in a narrower timeframe), whilst using the systematic and transparent methodology to identify, screen, appraise, and analyse evidence from relevant studies. The protocol for the study is registered under the International Prospective Register of Systematic Reviews (ID ref: CRD42020213431).

### Eligibility criteria

The full eligibility criteria for inclusion are described in Table 1a.

**Table 1:** **a** Study inclusion and exclusion criteria. **b** Search term and strategy outlined using PICO framework.

(a)			
Inclusion criteria		Exclusion criteria	
1. Qualitative, quantitative, and mixed design studies; including but not limited to descriptive; correlational; causal-comparative/quasi-experimental studies as well as case studies, ethnographic studies and narrative synthesis 2. Published between 2010-2020 to capture most recent publications 3. Studies including and reporting on participants with dementia (any type) 4. Studies looking at oral health practices and/or experiences and/or beliefs/views of older adults with dementia and their carers 5. Studies including and reporting on older adults living in community settings		1. Secondary research including systematic reviews, narrative reviews, or meta-analysis studies 2. Full-text not available 3. Not available in English 4. Grey literature 5. Studies including participants with general cognitive decline, but dementia diagnosis (any type) not specified	

(b)			
		OR	
**AND**	**Population** (Community Dwelling People with Dementia)	Dementia, Alzheimer’s disease, Alzheimer’s disease and related disorder*, elderly, aged	Carers, Care*
	**Interests** (beliefs, views and experiences)	Beliefs, views, experiences, practices, attitudes, perceptions, feelings, satisfaction, access, barriers, impact, *health knowledge, attitudes, practice	
	**Outcomes** (oral/dental health, practices and behaviours)	Dentistry, dental, oral health, dental care, *oral hygiene	
		Diet, fluoride, toothbrushing, toothpaste, denture cleaning	
	***Limits: 2010-2020, English***		

The study aims to draw on experiences, practices and beliefs/views of older adults living, diagnosed with dementia (and their carers) in community settings, therefore no direct comparator is required.; hence it is presented as PIO rather than PICO. Inormation (where available) was used to make comparisons against the type of settings (community living versus care homes), practice (self-care versus assisted care), carer type (formal vs informal).

### Search strategy and search terms

The search strategy was informed by library support services at King’s College London. Six databases were searched including Pubmed, Web of Science, OVID (Embase, MEDLINE [R] and PsycINFO) and Google Scholar. Searches were carried out in October 2020 and updated on 28 July 2021.

The search strategy included both keywords and National Library of Medicine’s MeSH (Medical Subject Headings). Given the limited research evidence in the field, broad search terms, identified in consultation with the library services/librarian, were necessary to capture evidence on the research questions. Search terms were combined using ‘OR’ and different groups of the PICO framework^21^ were combined using ‘AND’ for the final search outcome (Table 1b). Limits were applied to capture all relevant studies published in the last 10 years and in English language. In addition, manual citation tracking (backwards and forwards) for all included studies was conducted to identify additional relevant publications.

The search strategy was piloted ad refined in consultation with library services to ensure that key relevant papers were included.

### Screening and study selection

Screening of the articles retrieved was conducted in three stages: removal of duplicates, screening of title and abstracts and full-text screening. The screening strategy was piloted among reviewers to maintain consistency against the set eligibility criteria.

Titles and abstracts of all included studies were scanned by four reviewers in two pairs (SK, MA, SC, and AGP). Full texts obtained for the studies that met the inclusion criteria were then screened in duplication (SK and MA), with disagreements resolved by consensus within the research team. Clarification of ambiguous findings and/or incomplete data was sought by contacting the first authors of publications.

### Data extraction

Data extraction was carried out independently by two reviewers (SK and MA) using a customised data extraction table developed a priori and pilot tested for refinement. Differences were resolved in discussion or, if necessary, in consultation within the wider research team. Comparative data on participants without a dementia diagnosis and/or from non-community-dwelling residential settings were extracted for comparison, where available.

### Methodological quality assessment

The quality of all included studies was assessed independently and in duplication by two reviewers (SK and MA) using the Mixed Methods Appraisal Tool (MMAT), 2018.^22^ MMAT uses quality scoring criteria for five different study designs (i.e., qualitative, quantitative randomised control trials, quantitative non-randomised, descriptive, mixed) with approximately five criteria per study design category. It is validated for use and assessment of the quality, reliability and risk of bias across qualitative and quantitative, as well as mixed methods studies.^23^

### Data synthesis and analysis

Data were synthesised using a narrative disclosure given the heterogeneity of study design and data. Key characteristics of the studies are presented as an overall group (e.g., study year, date, country). Data are presented as subgroups pertaining to participant type (e.g., people with dementia, dementia type etc.), recruitment settings, and outcomes measured. Subgroup categorisation also includes information regarding key outcomes (beliefs/views, practices, experiences). It also includes analysis of longitudinal data and trends with regards to their dementia experience.

## Results

### Study selection

**Fig. 1:**
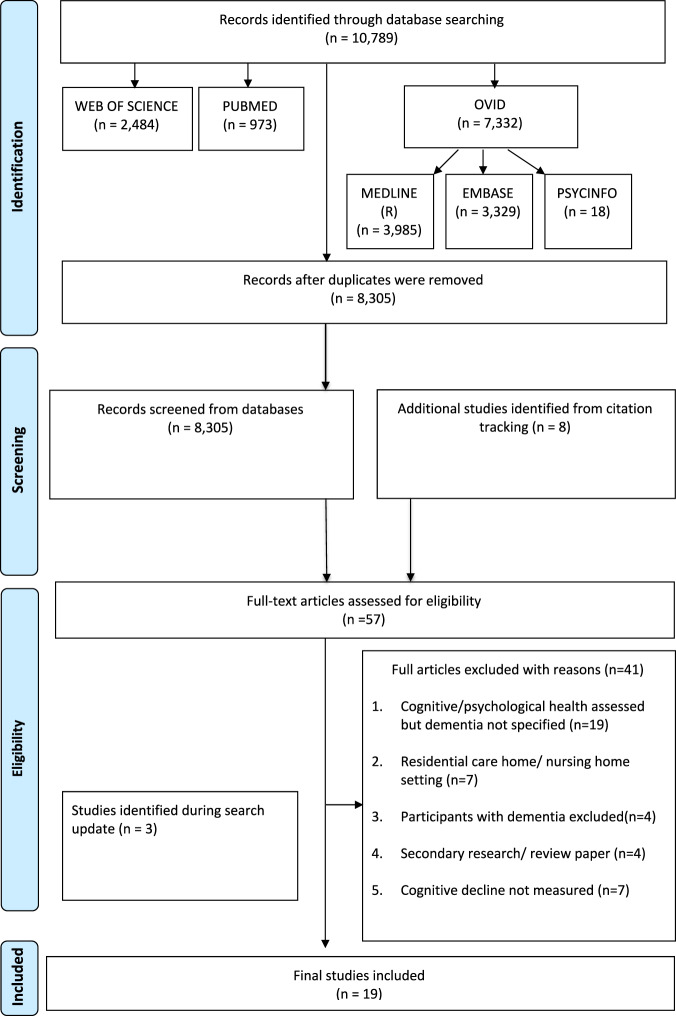
Prisma flow chart outlining literature search.

The electronic search resulted in 10,789 citations. Duplicates were removed to retain 8305 articles for abstract screening and an additional eight papers were identified from reference list screening. A total of 57 articles was shortlisted for full-text evaluation, of which, 16 met the defined inclusion and exclusion criteria and were retained for evaluation. Three additional papers were identified from the citation tracking, which resulted in a total of 19 papers representing 18 studies. The use of broad search terms to capture the breadth of research in the field while maintaining the minimum number of papers yielded many studies which did not meet the objectives of our review and were excluded during the initial screening. A PRISMA flow chart detailing the process of identification, database source, inclusion and exclusion of the studies is presented in Fig. 1.

### Study characteristics

Eighteen studies and 19 papers were included in the review, published between 2010 and 2020.^24–42^ Key study characteristics are presented in Table 2a. Seventeen papers were quantitative, which included six cross-sectional studies,^25,28–30,33,35^ two cross-sectional surveys,^31,34^ four cross-sectional, case-control studies,^26,38,40,42^ three case-control studies,^32,39,41^ two population-based cohort studies,^33,36^ one service evaluation,^37^ and one prospective study.^24^ Whilst two studies reported a mixed-method approach for data collection,^25,27^ only quantitative data were reported in the papers.

**Table 2:** **a** Table summarising the key characteristics of the included studies. **b** Table summarising the key findings reported by the studies reviewed.

(a)						
Author, date	Country	Participant type	Number of participants	Female/male	Age in years (Mean, SD)	Data collection tools
Araujo, 2020^39^	Brazil	People with dementia (*mild or moderate AD*) AND Informal carers (*family caregivers*)	Total = 102 Non dementia = 52 Dementia = 50	Non dementia: Female = 41 Male = 11 Dementia: Female = 31 Male = 19	Non dementia = 69.8 ± 1.0 Dementia = 72.6 ± 1.1	Clinical Dementia Rating (CDR) and Mini-mental State Examination (MMSE) scores calculated for a clinical diagnosis of AD Geriatric Oral Health Assessment Index (GOHAI) used for oral health-related quality of life measure Oral examination including Clinical Attachment Loss (CAL) and Probing pocket depth (PD), bleeding on probing (BOP), dental calculus (CL) and supragingival visible plaque (PL) measured for a clinical diagnosis of periodontists and disease severity Socio- demographic data collected.
Gao, 2020^38^	Hong Kong	People with dementia (*type and/or severity not specified*) AND People without dementia	Total = 228 Non dementia = 99 Dementia = 129	Non dementia: Female = 83 Male = 16 Dementia: Female = 97 Male = 32	Non dementia = 79.4 ± 6.7 Dementia = 80.9 ± 7.5	Demographic information (age and sex) and dementia status (yes/no) collected Questionnaire survey on oral hygiene practices of the participants, including daily toothbrushing frequency, difficulty in performing self-toothbrushing, and assistance in toothbrushing collected Clinical data on caries measured using decayed missing and filled teeth (DMFT); periodontal status using gingival bleeding, periodontal pocket, and loss of attachment recorded and oral hygiene status using Visible Plaque Index (VPI).
Emanuel, 2018^37^	England	People with dementia (*described as people with early signs of dementia and mild cognitive impairment, type and/or severity not specified*) And Formal carers (Nurses at Memory Assessment Services)	Non dementia =10 Dementia = 51	Not available	Not available	Questionnaire developed to gain insight into different aspect of general and preventive dental healthcare behaviour Also a separate questionnaire was developed for nurses at Memory Assessment Services.
Lexomboon, 2018^36^	Sweden	People with dementia (*including AD, dementia with Lewy-body, frontotemporal dementia, Parkinson’s disease with dementia, vascular dementia as well as other unspecified dementia type)*	Total = 34037	Female = 19,999 Male = 14,038	78–80	Data on medication use obtained from the Swedish Prescribed Drug Register, National Board of Health and Welfare Data on tooth extractions, dental restorations, and preventive procedures obtained from the Dental Health Register.
Campos, 2018^42^	Brazil	People with dementia (*mild AD*) AND People without dementia	Total = 32 Non dementia = 16 Dementia = 16 (Age–sex matched)	Non dementia: Female = 8 Male = 8 Dementia: Female = 8 Male = 8	Non dementia: 75.2 ± 4.4 Dementia: 76.7 ± 6.3	International Classification of Diseases, 10th version (ICD-10), DSM-IV, MMSE, and CDR scale used for dementia diagnosis and measure of decline Baseline information included stimulated salivary flow rate measured during clinical examination. Oral Health Related Quality of Life (OHRQoL) was evaluated using the validated Portuguese-language version of GOHAI Masticatory efficiency was evaluated using an artificial test material which patients were required to masticate for 40 cycles. The comminuted particles were collected in a paper filter, dried for 1 week at room temperature and passed through a sieving machine. The masticatory efficiency was calculated as the percentage weight of the comminuted material that passed through the 2.8-mm sieve OHRQoL and masticatory efficiency were re-evaluated after two months of using a new prosthesis.
Campos, 2017^40^	Brazil	People with dementia (*mild AD*) AND People without	Total = 32 Non dementia = 16 Dementia = 16 (Age-sex matched)	Non dementia: Female = 8 Male = 8 Dementia: Female = 8 Male = 8	Non dementia: 75.2 ± 4.4 Dementia: 76.7 ± 6.3	ICD-10, DSM-IV, MMSE, and CDR scale used for dementia diagnosis and measure of decline Clinical examination performed to assess/evaluate each subject’s teeth, removable prostheses, and presence of oral pathologies, such as ulcerations and prosthetic stomatitis Occlusal supports were recorded by using the Eichner Index; and, Kennedy Classification was also recorded for the partially edentulous subjects Sociodemographic data relating to educational level and monthly family income were also collected.
Campos, 2016^35^	Brazil	People with dementia (*mild AD*) AND Informal carers (*family caregivers*)	Total = 32 Non dementia (caregivers) = 16 Dementia = 16	Not described	Non dementia = 51.7 ± 11.1 Dementia = 76.7 ± 6.3	ICD-10, DSM-IV, MMSE, and CDR scale used for dementia diagnosis and measure of decline Sociodemographic and oral characteristics data collected Oral and clinical data objectively assessed by a single prosthetist dentist using DMFT rating Rise index used to score quality of prosthesis GOHAI Index 7 (validated Portuguese version) used to evaluate patient and caregiver assessment of oral health problems.
Luo, 2015^33^	Mainland China	People with dementia (*type and/or severity not specified*) AND people with mild cognitive impairment (MCI) AND People with normal cognitive function	Total = 3063 Non dementia/no cognitive impairments = 2398 Dementia = 121 MCI = 554	Total Female = 1664 Male = 1,399 Non dementia/ no cognitive impairments Female = 1279 Male = 1110 Dementia Female = 77 Male = 43 MCI Female = 308 Male = 246	Overall = 71.3 (SD 8.2) Non dementia/ no cognitive impairments = 70.0 (SD 7.7) Dementia = 80.9 (SD 7.4) MCI = 74.8 (SD 8.4)	Participants were interviewed face-to-face to collect sociodemographic data Presence or absence of dementia recorded using DSM-IV criteria. MCI defined according to Petersen’s criteria Zung Self-Rating Anxiety Scale was used to assess if participants had depressive or anxiety episode within the week prior to data collection. CDR and Brody Activity of Daily Living (ADL) scale used to obtain information on cognitive complaints and activities of daily living MMSE; Conflicting Instructions Task (Go/No Go Task); Stick Test; Modified Common Objects Sorting Test; Auditory Verbal Learning Test; Modified Fuld Object Memory Evaluation; Trail-making test A&B; RMB (Chinese currency) test conducted to measure domains of global cognition, executive function, spatial construction function, memory, language, and attention Oral health assessed using self-administered questionnaire with questions about the number of teeth missing and the medical history of oral health diseases and problems. The number of teeth missing (including third molars) counted and confirmed by the interviewers DNA extracted from blood or saliva. Apolipoprotein E genotyping conducted using the Taqman SNP method.
Lee, 2015^34^	USA	People with dementia (*type and/or severity not specified*) AND People with cognitive impairment, not dementia (CIND) AND People with normal cognitive function	Total = 329 Non dementia = 205 Cognitive impairment, no dementia (CIND) = 50 Dementia = 74	Female = 220 Male = 108	Non dementia = 77.47 ± 5.76 CIND = 80.88 ± 6.00 Dementia = 84.67 ± 7.43	A battery of neuropsychological measures used to assess verbal and visual memory, language, executive function, orientation, praxis, and reading ability. A proxy informant, usually a spouse or adult child, provided information about the participant’s cognitive function, functional limitations, medical history, and medications DSM-IV criteria used for diagnosis of dementia CIND was defined as mild cognitive or functional impairment reported by the participant or informant that did not meet criteria for dementia, or performance on neuropsychological measures that was both below expectation based on reading ability and educational and occupational history, and at least 1.5 SDs below published norms on any test within a cognitive domain. Final diagnosis was based on clinical judgement Clinical dental status assessment included number of missing teeth and number of decayed coronal and root surfaces.
Chu, 2015^32^	Hong Kong	People with dementia (*mild level of late onset Alzheimer’s disease)* AND People without dementia	Total = 118 Non dementia = 59 (age-sex matched) Dementia = 59	Dementia: Female = 47 Male = 12	Dementia = 79.8 ± 7.4 (Controls were matched for age and sex)	Unstimulated salivary flow rate measured using a saliometric assessment Clinical examination assessment included oral mucosal status, caries experience using the DMFT index and periodontal status using the Community Periodontal Index. No radiographs taken Toothbrushing practices, use of dental aids, difficulties in oral hygiene and personal data recorded using a questionnaire Medical history checked to ensure that they had no significant systemic diseases, such as valvular heart disease, prior to the oral examination The diagnosis and the stage of dementia recorded.
Chen, 2015^41^	USA	People with dementia (*type and/or severity not specified*) AND People cognitive impairment, not dementia (CIND) AND People with normal cognitive function	Total = 600 Non dementia = 492 CIND (cognitive impairment without dementia) = 57 Dementia = 51	Number not specified but explained that approximately two-thirds of the patients in each group were females.	All participants = 72.9 Non dementia = 71.6 CIND = 78.3 Dementia = 79.3	Medical history was either collected using a structured questionnaire or obtained from community group homes or day care programmes Clinical information obtained from dental chartings and radiographs included information on carious teeth and decayed retained roots. Additional information obtained included number of teeth; number of teeth with restorations; oral hygiene and gingival inflammation and use of a removable dental prosthesis Information on cognitive and functional status was also obtained from the dental records. Participants with a diagnosis of dementia according to International Classification of Diseases, 9^th^ version (ICD-9) or a diagnosis of Alzheimer’s disease, other types of dementia or chronic brain syndrome were included in the dementia group Information on patient capacity to perform oral hygiene was measured and reported by the dentist based on the caregiver’s assessment (for cognitively-impaired patients only), cognitive status, range of motion of the upper extremity and manual dexterity, oral hygiene at arrival and level of cooperation. Patient records provided information on other covariates including sociodemographic data, medications, physical mobility, disruptive behaviours, cooperation for dental care and language impairment.
Del Brutto, 2014^31^	Ecuador	People with dementia (*type and/or severity not specified*) AND People without dementia (including those with depression) Data presented in relation to number of missing teeth.	Total = 274	Female = 162 Male = 112	69.6 ± 7.7	Oral exam included an emphasis on the number of remaining teeth A questionnaire used to allow participants to self-rate their oral hygiene as poor, fair or good, on the basis of questions regarding regular tooth brushing, use of mouthwash antiseptics and dental floss, and periodic preventive visits to the dentist Dementia diagnosed using Legane ´s Cognitive Test. Cognition was assessed with the Spanish version of the Montreal Cognitive Assessment (MoCA) test.
Cicciu, 2013^30^	Italy	People with dementia (*AD, severity not specified)*	Total = 158	Female = 101 Male = 57	74.37 ± 5.38	General demographic data was collected Clinical data collected using DMFT score and periodontal disease check for parameters including probing depth, bleeding and tooth mobility. Orthopantomography X ray exam taken. A self-reported Italian version of Oral Health Impact Profile (OHIP-14) used to capture the participants’ perception of their own oral health within 3 months prior to the test.
Chen, 2013^29^	USA	People with dementia (*type and/or severity not specified*)	Community = 51 Assisted living = 18 Nursing home = 501	In community setting: Female = 35 Male = 16 Assisted living: Female = 9 Male = 9 Nursing home: Female = 363 Male = 138	In community setting = 79.3 (SD 8.0) Assisted living = 80.9 (SD 12.6) Nursing home = 82.6 (SD 9.6)	Medical history was collected using a structured questionnaire Comprehensive oral assessment, including oral hygiene gingival inflammation, caries assessment, oral mucosal lesions, and denture assessment completed for study participants. Full mouth radiographs were also taken Classification of Diseases, 9th Revision, or 331.2^26^ or a diagnosis of Alzheimer’s disease, other types of dementia, or chronic brain syndrome recorded in their medical history were considered to have dementia Cognitive assessment was based on a set of subjective approaches including (1) administering part of the MMSE; (2) asking caregivers about the cognitive status of the patients; (3) assessing cognitive status through verbal communication; and (4) asking the patient to repeat and/or demonstrate clinical instructions. The ability to co-operate as well as to communicate oral health needs also assessed A set of subjective and objective approaches was collectively used to assess capacity to perform oral hygiene care Physical mobility was also evaluated using a 4-level scale.
Lee, 2013^28^	USA	People with dementia (*mild, type not specified)* AND People cognitive impairment, not dementia (CIND) AND People with normal cognitive function	Total = 226 Non dementia = 169 CIND (cognitive impairment without dementia) = 38 Mild dementia = 19	Total: Female = 149 Male = 77 Non dementia: Female = 109 Male = 60 CIND: Female = 25 Male = 13 Mild dementia: Female = 15 Male = 4	Non dementia = 77.4 ± 5 CIND = 80.6 ± 5.4 Mild dementia = 83.9 ± 7.9	Oral health-related quality of life measured using GOHAI Cognitive function was assessed using comprehensive neuropsychological test battery and DSM-IV were used for diagnosis of dementia Socio-demographical variable and medical conditions measured. Depressive symptoms were assessed using the Geriatric Depression Scale. Clinical dental status measured by assessing number of decayed coronal and root surfaces, number of missing teeth, plaque index (for oral hygiene measure), and the mean pocket depth (for periodontal disease measure).
Srisilapanan, 2013^27^	Thailand	People with dementia (AD, *vascular dementia, alcohol dementia and with dementia in other forms in mild, moderate and severe stages*)	Total = 69 (All patients had access to dental services delivered in conjunction with the memory clinic)	Total = 69 Female = 43 Male = 26	75.5 ± 7.0	Thai version of the Mini-Mental State Examination (MMSE-Thai 2002) was used as a cognitive testing instrument. Sociodemographic data and functional ability were obtained from the hospital medical records Sirindhron National Medical Rehabilitation Center-Functional Assessment was used to measure functional assessment. The assessment of the ability to perform oral care was done by an interview of patients using a modification from the assessment of activities for daily living. The evaluation of dental caries was done using the DMFT based on WHO criteria. Periodontal status was measured by using the Community Periodontal Index. Denture status was evaluated as worn, had denture(s) but did not wear, never had denture(s). Functional teeth were defined as natural teeth that could be used for biting and chewing. The judgement was based primarily on the clinical examiner with additional information from direct interview.
Ribeiro, 2012^26^	Brazil	People with dementia (*AD in mild, moderate and severe stages)* AND People without dementia	Dementia = 30 Non dementia = 30	Female = 23 Male = 7	67.80 ± 5.45 Non dementia = 66.0 (59.0– 81.0) Dementia = 78.0 (68.0–89.0)	ICD-10, DSM-IV, MMSE, and CDR used for diagnosis of AD. Volunteer-reported oral health data collected using GOHAI Demographic and oral characteristics were assessed, including the number of natural teeth; DMTF; OHI; removable prosthesis conditions; and oral pathologies.
Syrjala, 2012^25^	Finland	People with dementia (*AD, vascular dementia and other types of dementia [including dementia with Lewy bodies or other types or unknown types]; all in mild, moderate or severe stages*) AND People without dementia	Total = 354 Non dementia = 278 AD = 49 Vascular dementia = 16 Other types of dementia = 11	Female = 253 Male = 101	82	Diagnosis of dementia made according to the DSM-IV criteria as well as, criteria presented by McKeith. Severity of dementia rated according the DSM-III-R guidelines of the American Psychiatric Association. A geriatrician and trained nurse carried out structured clinical examination and interview. Participants were interviewed on their health, health behaviour and social life. Overall physical and mental status as well as drugs used were also reviewed. History of cognitive decline obtained by interviewing the participants and their relatives and examining medical records. Oral clinical examination and structured interview on oral health habits conducted.
Hatipoglu, 2011^24^	Turkey	People with dementia (*AD; mild, moderate and severe stages*) AND People without dementia	Total = 78 Non dementia = 47 (age–sex matched) Dementia = 31	Non dementia: Female = 28 Male = 19 Dementia: Female = 17 Male =14	Non dementia = 65.32 ± 6.95 Dementia = 67.61 ± 9.14	Instrumental activities of daily living, Index of activities of daily living scales and MMSE used to assess cognitive and function of those with dementia. DMFT scores calculated and used to assess the dental health status. Oral hygiene status evaluated using the plaque index and prosthesis plaque index. Mucosal findings including any ulcerations, hyperaemia, and hyperplasia noted if present. The presence of any dentures and any denture- related findings, such as denture stomatitis and removal of dentures evaluated.

(b)			
Author and date	Aims	Key findings	Conclusions
Araujo, 2020^39^	To test the hypothesis that periodontitis is associated with Alzheimer’s Disease (AD) and search whether periodontal and other variables would negatively impact the oral OHRQoL perception.	Cases had fewer teeth and greater clinical attachment loss (CAL) than controls. AD patients presented more advanced periodontitis, compared with controls. Number of teeth (mean ± SD) in the case group = 11.5 ± 6.2 compared to 20.8 ± 6.1 in the control group. Mean CAL in the case group = 4.6 ± 0.3 and in the control group = 3.1 ± 0.2 Periodontitis was a variable most likely associated with AD. Most individuals were females. However, men had higher prevalence of periodontitis (78.9% vs. 61.3%, *p* = 0.005) and exhibited a more severe periodontitis with deeper PD and greater CAL mean values (respectively 3.8 ± 1.8 vs. 3.1 ± 1.1, *p* = 0.005 and 5.3 ± 2.1 vs. 4.1 ± 1.5, *p* < 0.001). The mean GOHAI score in the case group was superior to that of the control group, which corresponds to a positive self- perception of their oral health-related quality of life. Mean score for the case group = 32.6 ± 2.8 and control group = 30 ± 3.8 Whereas 100% of patients in the AD subgroup mild reported no discomfort during chewing, the proportion of those responders in the moderate category dropped to 75%. While 88.5% of patients in the AD subgroup mild responded being always satisfied with their smile appearance, that prevalence reduced to 50% in the subgroup moderate. Other items of the GOHAI are not described.	Periodontitis is associated with Alzheimer’s disease, but not with patients’ perceived oral health-related quality of life. AD patients, although having fewer teeth and severe periodontitis, have more positive GOHAI scores, when compared with the control group. People with Moderate AD present worse oral status and reports more negative impact compared to those with mild disease
Gao, 2020^38^	To compare the caries, periodontal status, and toothbrushing practices of Chinese elderly people with and without dementia.	The mean DMFT score of the elderly with dementia was significantly higher than that of the elderly without dementia (22.5 vs. 19.2, *p* = 0.041) Almost all the elderly people with dementia (98%) had gingival bleeding. Around two-thirds of them (64%) had periodontal pockets. More than half of them (54%) had teeth with a loss of attachment of at least 6 mm. People with dementia had a significantly higher level of visible plaque than people without dementia (77% vs. 63%). More than half of the participants with dementia reported difficulties when performing toothbrushing, while only a few reported so in nondementia group (57% vs. 8%, *p* < 0.001). More elderly people in the dementia group required caregiver-assisted toothbrushing than those without dementia. More elderly people in the dementia group required caregiver-assisted toothbrushing than those without dementia (17% vs. 3%, *p* = 0.001). Four elderly people (3%) with dementia stated they did not brush their teeth daily, whereas all elderly without dementia performed toothbrushing every day. More than one-third (36%) of the elderly with dementia and 44% of the elderly without dementia had dental visits within one year, and the difference was not statistically significant (*p* = 0.241).	Compared with elderly people without dementia, Chinese elderly people with dementia had more caries experience and poorer oral hygiene. More elderly people with dementia experienced difficulties in toothbrushing, but only a few of them had received assisted toothbrushing by the caregiver.
Emanuel, 2018^37^	To examine and record current access to primary care dental prevention advice and care, for patients with a recent diagnosis of early-stage dementia.	The results show that oral health is important to patientsattending MAS with 80% either registered or regularly attending a dental practice. Participants had early dementia and mild cognitive impairment. About half of all patients attended for regular hygienist sessions. Most patients did not receive dietary advice or oral hygiene instruction, nor were offered additional fluoride supplementation When asked to provide comment on their current dental care, 55% (28) gave a positive comment, 18% (nine) a negative comment. When asked to self-report their perceived level of oral health, 10% (five) reported it as excellent, 63% (32) reported it as good: Patients who were asked to rate their own oral health may have provided a very subjective opinion of their ‘good’ oral health. The MAS nurses did report a higher frequency of being offered prevention advice by their dentist or hygienist with 70% reporting having been given advice against the 20% of patients reporting having received advice. All MAS staff were unaware of the current Delivering Better Oral Health toolkit, this suggests that they may benefit from further detailed education in service provision for them to disseminate to their patients. A high number of MAS nurses believed that oral health of dementia patients was clinically very important.	Despite a high attendance rate reported by patients with a recent diagnosis of dementia, there is a concern that the necessary prevention advice needed for these patients is not necessarily being provided. MAS nurses and other care staff could be trained on how to advise patients on preventative oral healthcare at the point of first contact. Emphasis should be made that oral health is an integral part of overall health and as such, can prevent additional suffering from oral problems.
Lexomboon, 2018^36^	To describe the association between the number of xerostomic medications used and tooth loss and restorative and dental preventive treatment in a population of persons with dementia.	The average number of xerostomic medications was positively associated with tooth extraction (IRR 1⁄4 1.07; P 1⁄4 .007). Compared to persons who did not use any xerostomic medication (reference group), the average number of extractions was 2.7%, 11.5%, and 39.9% higher among per- sons using up to 1, up to 3, and more than 3 xerostomic medications (IRR 1⁄4 1.03, 1.11, and 1.40; P 1⁄4 .042), respectively. However, the risk for having new dental restorations and receiving preventive procedures did not differ between groups (P 1⁄4 .555 and P 1⁄4 .827).	This study found a positive dose-response relationship between xerostomic medication use and increased risk for dental extraction among persons with dementia. No association was found between xerostomic medication use and dental restorations or preventive procedures. This study highlights the importance of careful consideration when prescribing xerostomic medications in people with dementia, and the need for regular and ongoing dental care for these patients. Findings show that xerostomic medication can contribute to poorer oral health in a longitudinal and dose-response manner.
Campos, 2018^42^	To compare the masticatory function and Oral Health Related Quality of Life (OHRQoL) of elderly people with mild AD with a control group of elders without cognitive impairment before and after oral rehabilitation with removable partial dentures (RPDs) and/or complete dentures (CDs).	The salivary flow rate was lower (*p* < 0.05) in the AD group (0.73 ± 0.52) than in the control group (1.19 ± 0.65). Masticatory efficiency was impaired in patients with AD compared with the control group both before and after prosthetic treatment (*p* < .05). Both groups benefited from treatment, which increased the masticatory efficiency values (*p* < 0.05). After rehabilitation with new removable prostheses, both the AD and control groups presented higher GOHAI values, demonstrating improvement in OHR- QoL (*p* < 0.05). However, comparisons between groups showed that GOHAI values were significantly higher for the AD group than for the control group in both analysed times (*p* < 0.05), showing less impact of oral health on quality of life of patients with AD.	Although the dental condition between the AD and control groups was matched, masticatory efficiency was significantly lower in patients with mild AD before and after insertion of the new prostheses. Individuals with AD had lower salivary flow compared to controls. They also had reduced masticatory efficiency and less improvement in OHRQoL compared with control.
Campos, 2017^40^	To objectively describe masticatory performance in elderly with AD, compared to a cognitively healthy control group.	No group differences were found in educational level (AD, 4.31 ± 5.41 years; control, 4.05 ± 3.20 years), monthly family income (AD, 2.41 ± 2.27; control, 2.69 ± 2.66), number of remaining teeth (AD, 4.31 ± 6.82; control, 3.94 ± 6.54). Compared to controls, AD subjects had decreased MMSE values and masticatory performance. There was a moderate negative correlation between masticatory performance (X_50_) and cognitive parameters (Pearson’s *r* = −0.69, *p* < 0.001; power *β* = 0.83). Thus, greater MMSE scores were associated with lower X_50_ values, indicating greater chewing deficits.	Mild AD was associated with impaired chewing function.
Campos, 2016^35^	To test the hypothesis that patients and their caregivers present a distinct pattern of responses on perceptions of OHRQoL in mild AD by collecting GOHAI ratings and correlating these scores with objective clinical findings.	The absence of teeth was a remarkable characteristic in AD patients. The majority of patients were completely edentulous and wore dentures in both dental arches. Partially edentulous patients retained very few teeth, averaging fewer than five. The caregiver group was mostly fully dentate and the partially dentate have few missing teeth. In the AD group the mean Rise index was low for all issues evaluated, demonstrating that prostheses were considered as grade I unsatisfactory. Agreement between patient and caregiver GOHAI scores was very good (0.8 ≤ *κ* ≤ 1.0) for psychological domain and for items 3, 6, 7, 8, and 11; good (0.6 ≤ *κ* ≤ 0.79) for total score and items 4, 5, 9, 10, and 12; moderate (0.4 ≤ *κ* < 0.59) for physical domain and items 1 and 2. AD patients and their caregivers reported similar GOHAI total scores. However, some GOHAI items showed poor correlations between AD patient and caregiver ratings: were related to the physical domain, involving speech and mastication, and sensibility to hot or cold of AD subjects The study did not find correlation between the objective measures of prostheses quality obtained from AD patients and their caregivers.	Mild AD patients assess and self-report their OHRQoL similarly to their main caregivers and those reports do not correlate with prostheses quality clinical measures. GOHAI index obtained from individuals with mild AD and their caregivers were similar. However, some GOHAI items, such as those related to speaking, biting, and amount of food eaten, showed poor correlations between AD patient and caregiver ratings. With regards to prosthesis quality, AD patients’ dentures were slightly unsatisfactory according to Rise criteria.
Luo, 2015^33^	To examine the relationship between tooth loss and cognitive function among a sample of older Chinese population, in which the cognitive function of each participant was determined by neurologists through a series of neuropsychological tests.	The mean number of teeth missing was 10.2 (SD = 9.7) for all the participants. Participants with dementia had a significantly higher number of teeth missing (mean 18.7) than those with mild cognitive impairment (MCI) (mean 11.8) and cognitive normal (mean 9.3). Sixty per-cent of participants with dementia had lost more than 16 teeth. Number of teeth missing was associated with cognitive impairment. Subjects with a loss more than 16 teeth reported higher prevalence of dementia. Participants who lost >16 teeth had an OR (odds ratio) for dementia of 3.65 (95% CI 2.75–4.86), and had an OR for MCI of 1.42. After adjusting for confounding variables, tooth loss of >16 were significantly associated with dementia with an OR of 1.56.	In conclusion, the number of teeth missing was significantly associated with severe cognitive impairment. Poor oral health might be considered as a related factor of neurodegenerative symptom among older Chinese population.
Lee, 2015^34^	To examine the relationship between cognitive impairment and dental care utilisation among older adults in the United States.	Individuals with normal cognitive function were younger and more likely to report a higher level of social support than individuals with CIND and dementia (normal—5.52 ± 2.50, CIND-4.38 ± 2.52 and dementia- 3.37 ± 1.77). As the level of cognitive function worsened, individuals were less likely to have had recent dental visits and regular dental checkups. The time since the most recent dental visit was longer for individuals with dementia compared to those with normal cognitive function. Those with the most recent dental visit >5 years ago: normal- 10, CIND-2, Dementia-2. Perceived confidence in network and the presence of dental insurance were associated with less time since the last dental visit (perceived confidence in network: normal-6.78 ± 2.3, CIND-5.84 ± 2.37, Dementia—3.86 ± 1.96) Individuals with CIND and dementia were less likely to have regular dental visits than individuals with normal cognitive function (Those with regular dental visits, 2 or more times a year: Normal- 117, CIND-19, Dementia 9) Greater perceived social network and the presence of dental insurance were associated with more frequent dental visits. A higher number of missing teeth was associated with less frequent dental visits.	Less dental care utilisation may contribute to the oral health problems often observed among individuals with dementia. Efforts to increase use of dental care should consider including cost-effective options for dental insurance. In addition, educating formal and informal caregivers on the importance of dental care may be beneficial, as these individuals are in the best position to facilitate dental care for individuals with dementia.
Chu, 2015^32^	To compare toothbrushing habits, unstimulated salivary flow rates and oral health status of elderly Hong Kong Chinese with and without dementia.	There were 63% people from the dementia group and 5% people from the control group who had difficulties with oral hygiene practice. The main problems encountered by the dementia participants were forgetfulness (forgetting to brush or forgetting that he/she had already brushed) (73%), unwilling to brush (35%), inability to brush (lack of dexterity and the lack of an assistant to help perform brushing) (22%). The mean unstimulated salivary flow rates (ml/min) of the dementia and control groups were 0.30 ± 0.17 and 0.41 ± 0.28, respectively (*p* = 0.043). Most dementia participants (95%, *n* = 56) had a healthy oral mucosal status. The caries experience (mean DMFT ± SD) was 22.3 ± 8.2 for the dementia group and 21.5 ± 8.2 for the control group (*p* = 0.585). More people in the dementia group received assistance in brushing than did people in the control group (31% vs 5%; *p* < 0.001). Fewer people in the dementia group brushed at least twice daily than in the control group (67% vs 83%; *p* = 0.045). No association was found between the DMFT score and regular toothbrushing. No one (0%) suffering from dementia and only one (2%) participant without dementia had a healthy periodontal status.	In this study, there were fewer elderly Chinese with dementia than without who practiced toothbrushing twice daily. Although their resting salivary secretion was reduced, their caries experience and prevalence of advanced periodontal disease were found to be similar to those without dementia.
Chen, 2015^41^	To investigate whether oral self-care function mediates the associations between cognitive impairment and caries severity in community- dwelling older adults.	A significant proportion of the patients with cognitive impairment (43% in CIND group and 66% in dementia group) needed supervision/help to maintain oral hygiene, which was significantly higher than that of the group without any cognitive impartments (*p* < 0.001). On average, patients in the CIND and dementia groups had 6.1 and 5.5 teeth with caries or retained roots at arrival, respectively, which were significantly higher than 3.3 in the group without cognitive impairments (*p* < 0.001). Participants in the CIND and dementia groups had 1.7 (95% CI: 1.13, 2.46) and 1.8 (95% CI: 1.23, 2.70) times more likely to have a carious tooth or retained root, respectively, than the group with normal cognitive function. While the capacity to perform oral hygiene was adjusted for, this association was no longer significant. On the other hand, participants who lost their ability to perform oral hygiene had 1.7 (95% CI: 1.15, 2.44) times greater risk of having a carious tooth or retained root than those who were self-sufficient.	Cognitive impairment and capacity to perform oral hygiene were both associated with the number of carious teeth or retained roots. Cognitive impairment became insignificant when oral care capacity was adjusted for, indicating capacity to perform oral hygiene care mediates the association between cognitive impairment and dental caries severity among cognitively-impaired patients.
Del Brutto, 2014^31^	To assess whether edentulism associates with cognitive impairment in elders living in rural Ecuador.	Persons with <10 remaining teeth (*n* = 116) have significantly lower MoCA scores (17.2 ± 4.6) than those with >10 teeth (*n* = 158) (MoCa score 19.4 ± 4.5), after adjusting for demographics, cardiovascular risk factors, depression and dementia. Self-rated poor oral health was more prevalent among persons with <10 teeth but did not correlate with MoCA scores. A poor oral health (92% poor OH in the <10 teeth group compares to 1% poor OH in those >10 teeth) was more prevalent among persons with severe edentulism. The group of persons with< 10 remaining teeth was older, 13% with dementia and less educated than the group with >10 teeth which included 6% with dementia.	Severe edentulism is associated with a poorer cognitive performance in elders living in rural Ecuador.
Cicciu, 2013^30^	To describe the relationship between caries, periodontal disease frequency and quality of life in AD patients.	The DMFT index was 23.56 ± 2.78, with 6.89 ± 3.02 being Decayed, 14.04 ± 4.39 Missing and 2.62 ± 1.79 Filling teeth. The Plaque Index (PI) registered was of 70.86 ± 13.76%. The ratio between diagnosis of periodontal disease and impact on quality of life was significant in individuals with periodontitis (*p* < 0.001) and missed filled teeth. Gingival bleeding, and probing depth > 4 mm were associated with intensely negative impact on quality of life (*p* = 0.013, p < 0.001, and *p* = 0.012 respectively). The absence of more than 2 molar teeth increases the chewing inability, decreasing the patient quality of life. Comparing the ages we observed also that the patients with more missing teeth were older than the ones with a more complete, but unhealthy, dentition, correlating the incidence of oral diseases with the progressing of AD.	It was observed a correlation between the age and the high index of pathologies analysed, due to the progressive nature of the disease. The decrease of cognitive functions caused a deterioration of oral hygiene procedures: tooth brushing was irregular in this sample of AD patients. Concepts of health and disease determined by clinical diagnostic criteria may influence the assessment of the impact of periodontal disease on Alzheimer’s quality of life.
Chen, 2013^29^	To compare oral health in dementia patients living in different environments.	Oral health was poor in study participants, regardless of their residential status. Nearly 30% of Nursing home (NH) residents lost all their natural teeth, almost double those living in community or assisted living facilities. 60% of the community-dwelling patients had 9 to 24 teeth, significantly higher than that of NH residents. Living environment was not associated with oral health measures, indicating oral health had declined in dementia patients before they were placed into NH. More assisted living residents and NH residents had impaired physical mobility and required help/supervision in performing oral hygiene care. (walk independently (%); community group: 52.1, Assisted leaving: 17.6, nursing home: 12.4) Capacity to perform oral hygiene (%) Self-sufficient: Community: 33.3, assisted living: 27.8, nursing home: 18.0 Nearly half of the elderly living in the community and NH wore a dental prosthesis (*p* = 0.78) at arrival whereas only 38.9% of assisted living elders had a dental prosthesis. NH residents tended to be older than assisted living residents and those living in community On average, NH residents had 10.5 chronic medical conditions, significantly higher than assisted living residents and community- dwelling elders.	Oral health was poor in older adults with dementia, regardless of their residential status. After adjusting for other factors, residential status was not significantly associated with oral health in participants with dementia, indicating oral health had declined in these individuals before they were admitted into NH. Effective and aggressive intervention strategies should be established to appropriately address oral health needs in older adults with dementia even before they are placed into LTC facilities.
Lee, 2013^28^	To examine the relationship between cognitive function and self-reported OHRQoL in community dwelling older adults.	Participants with normal cognitive function had higher GOHAI total scores (mean: 55.1), indicating better oral health-related QoL, than participants with cognitive impairment without dementia (CIND) (mean: 52.3) and mild dementia (mean: 51.0). The difference remained significant after controlling for covariates including socio-demographics, health status, comorbidity, and clinical dental status. The number of decayed coronal surfaces was the only significant clinical predictor; alone it accounted for about 19% of the GOHAI total score. Participants with normal cognitive function had higher GOHAI total scores than participants with CIND (*β* = −0.31, *p* = 0.02) and mild dementia (*β* = −0.38, *p* = 0.02). Individuals with more decayed coronal surfaces reported lower GOHAI total scores (β = −0.31, *p* = 0.04).	Oral health-related QoL, as measured by the GOHAI, was better among those with normal cognitive function compared to those with CIND and those with mild dementia in the population studied.
Srisilapanan, 2013^27^	To investigate the oral health of patients with dementia and examine the association between the type and severity level of dementia on their dental caries status.	The group with the highest prevalence of dementia was 70 to 79 years old. Most of the patients (56.5%) had moderately severe dementia. The assessment of functional ability showed that more than half (66.7%) were independent with minimal assistance. Almost half (49.3%) were able to perform oral care by themselves. Most of the patients with dementia had at least some natural teeth. The proportion of dementia patients who wore dentures was equal to those reported that they had never worn denture. Almost 20% reported that they had dentures but never wore them. Approximately one-third had calculus (34.0%). About one-third (30.2%) had a pocket depth of 4–5 mm. Dementia severity was the only characteristic which showed a significant difference in dental caries experience (*p* = 0.009).	Dementia patients who attended the memory clinic had considerably better oral status compared to the national data. Dementia severity was the only characteristic that showed a significant difference in dental caries experience.
Ribeiro, 2012^26^	To describe the oral health of elderly people diagnosed with AD.	The GOHAI scores were similar and considered moderate for both groups (32.0 in the control group and AD group: mild-33.0, moderate-32.5, severe-34.0). Subjects who presented a fewer number of natural teeth and OHI values than the controls (mean scores; control group: 13.5 teeth and AD group: mild—4.0, moderate— 0.0, Severe 0.0). OHI in control group: 2.2, AD group: mild- 4.0, moderate- 5.0, Severe 7.5) Comparisons between the GOHAI scores of subjects with AD of different disease stages and the controls revealed that the GOHAI scores were similar. The GOHAI scores were considered high only for subjects with AD in the severe stage of the disease (34.0); the number of natural teeth was lower for subjects with AD in the moderate and severe stages of the disease. A similar trend was observed for the DTMF values, which were higher in subjects with moderate and severe AD. (average DMFT in AD groups: mild- 27.0, 28.0, 28.0 and 25.5 in control). Analysis of removable prosthetic conditions between subjects with AD and healthy volunteers revealed a significant association between the presence of oral pathology and AD. Prosthetic stomatitis was the most commonly observed lesion, with a prevalence of 60%.	Elderly subjects with AD had poorer oral health than those without the disease. Despite the positive self-perception of their oral health, the oral health of subjects with AD tended to decline as their disease progressed.
Syrjala, 2012^25^	To study the association between diagnosed dementia and oral health, focusing on the type of dementia, among an elderly population aged 75 years or older.	Persons with Alzheimer’s disease and persons with other types of dementia had an increased likelihood of having carious teeth, teeth with deep periodontal pockets and poor oral and denture hygiene, compared with non-demented persons. The proportion of dentate persons among patients with Alzheimer’s disease was 37%; with vascular dementia, 31%; and with other types of dementia, 27%. Among patients with Alzheimer’s disease, the proportion of persons with poor oral hygiene was 78% and with poor denture hygiene, 75%. Among patients with vascular dementia, the proportions were 60% and 73%, and among patients with other types of dementia, 67% and 63%, respectively.	Among the elderly aged 75 years or older, patients with Alzheimer’s disease or other types of dementia are at increased risk of poor oral health and poor oral hygiene.
Hatipoglu, 2011^24^	To evaluate the oral health status in patients with AD, and the association of the disease severity with the oral findings.	DMFT scores (AD group: 24.19 ± 6.8, Control group: 19.68 ± 9.5), number of the present teeth (AD group: 5.07 ± 7.7, Control group: 10.55 ± 10.6) and maxillary and mandibular dentures were similar in the patients with AD and in the control group. Oral hygiene status and maxillary and mandibular denture status were similar in the patients with AD and in the control group. However, the patients with AD were found to have denture- related stomatitis (AD group:13/22, Control: 2/27) due to the lack of denture removal at night (Not removing mandibular denture at night; AD group: 14/22, Control: 6/26), Not removing maxillary denture at night; AD group: 15/22, control: 6/27) In this study, MMSE scores were significantly correlated with DMFT, PT, oral hygiene status, denture removal at night and related stomatitis. In this study, decreased cognitive functions in AD patients were found to be the result of poor oral hygiene habits. Multiple factors such as continuous denture wear, trauma from denture and poor oral hygiene have been reported to affect dental health in the patients with dentures. Denture stomatitis is one of the most common findings	The results of this study indicate that oral hygiene status is closely related with cognitive functions of the patients with AD. Especially, denture-related problems may be obvious due to impaired denture care in these patients. Here we believe that these patients should be consulted regularly with a dentist and care providers should be instructed the importance of oral hygiene and denture care.

*CDR* Clinical Dementia Rating, *MMSE* Mini-mental State Examination, *AD* Alzheimer’s Dementia, *GOHAI* Geriatric Oral Health Assessment Index, *CAL* clinical attachment loss, *PD* Probing pocket depth, *BOP* bleeding on probing, *CL* dental calculus, *PL* visible plaque, *DMFT* decayed missing and filled teeth, *VPI* Visible Plaque Index, *ICD-10* International Classification of Diseases 10th version, *DSM-IV* Diagnostic and Statistical Manual of Mental Disorders 4th Edition, *OHRQoL* Oral Health Related Quality of Life, *ADL* Brody Activity of Daily Living, *CIND* cognitive impairment, nodementia, *ICD-9* InternationalClassification of Diseases, 9th version, *MoCA* Montreal Cognitive Assessment, *OHIP-14* Oral Health Impact Profile, *OHI* Oral Health Index, *MAS* Memory Assessment Services, *MCI* mild cognitive impairment, *CPI* Community Periodontal Index.

Across all included studies, the sample size ranged from 32,^35^ to 34,037.^36^ The papers presented a wide geographical representation with four studies from the United States of America (USA),^28,29,34,41^ and two in Hong Kong.^32,38^ Three studies across four publications were from Brazil,^35,39,40,42^ and one each from China,^40^ Finland,^32^ Ecuador,^31^ Thailand,^27^ Turkey,^24^ Italy,^30^ Sweden,^36^ and England.^37^

Participants were recruited from a range of community settings including those from dementia networks,^26,35,40,42^ university hospitals or public health institutes,^24,30,39^ daycare centres,^29,38^ memory assessment clinics,^27,37^ national registry or local communities,^25,28,31,33,34,36^ or a combination of the above. ^41^ Out of the four studies conducted in Brazil three,^35,40,42^ included participants from the same community settings, with two papers that included the same group of participants.^40,42^ The papers did not detail the living arrangements sufficiently to distinguish between older adults living in their own homes or in supportive housing facilities.

Fourteen papers directly included older adults with a dementia diagnosis and only five studies included caregivers in various capacities,^26,29,35,37,39^ one of which involved formal caregivers, as represented by nurses from Memory Assessment Clinic.^37^

Whilst a majority of studies used standardised tools to measure certain aspects of oral health status, there was limited information collected on oral health beliefs/views, care experiences or self-care practices. Dental caries was the most frequently recorded clinical disease measure, which was assessed using the DMFT (Decayed, Missing, and Filled Teeth) index,^43^ whilst oral hygiene, gingival bleeding, plaque levels and pocket depth were used to assess periodontal diseases. The International Statistical Classification of Diseases and Related Health Problems (ICD-10) and Diagnostic and Statistical Manual of Mental Disorders, 4th edition (DSM-IV) were the most common indicators of dementia diagnosis, whereas, the Mini-Mental State Examination (MMSE) tool,^44^ was the most common proxy measure of cognitive decline. Quality of life was most frequently reported using the General Oral Health Assessment Index (GOHAI).

### Study participants

The age range of participants with dementia ranged from 72 to 82 years old, most of whom were female. Information regarding the type and severity of dementia in study participants was reported in most studies, but with varied detail (Table 2a).

### Oral and dental disease

Most of the studies were clinically focused and reported on the oral and dental disease status of those with dementia.^24–27,29,31–36,38,41^

Generally, across all studies, the findings suggest that people with dementia had worse oral and dental health compared to those without dementia or their carers. The only exception to this was the study by Srisilapanan and Jai-Ua,^27^ where the provision of frequent dental appointments was made for older adults attending memory services for dementia. Access to dental care had a significant influence on oral health improvement for this study population, who presented with a similar DMFT score to the national average. Therefore, the fact that health systems for those with dementia had made dental care accessible, their oral health was not worse than the general population.

Generally, the absence of teeth, periodontal disease risk and dental caries were common. Luo et al.^33^ found that people with dementia had more teeth missing than those with moderate or no cognitive impairment, even after adjusting for confounders. While Gao et al.^38^ found no significant difference in some of the periodontal disease parameters (i.e., gingival bleeding, periodontal pockets and loss of attachment), the dementia group still had significantly higher levels of visible plaque and caries compared to those without dementia. Similarly, Del Brutto et al.^31^ reported that oral health was worse in people with dementia, which was directly correlated to the number of missing teeth. People with more than ten missing teeth presented with worse oral health. Arajulo et al.,^39^ looked at both number of missing teeth and periodontal disease status in older adults with dementia and found that this group had worse periodontitis and more missing teeth compared to their carers. They also frequently reported discomfort while chewing. Similar findings were reported by Campos,^40^ who showed that people with dementia had a decreased masticatory performance, which was negatively corelated to their cognitive function. The study found that those with more severe dementia-related cognitive decline, also had a lower masticatory function, which impacted their chewing capacity. Therefore, older adults with dementia, even at early stages of cognitive decline, appear to be at a higher risk of oral health deterioration.

The use removable prosthesis like dentures to replace missing tooth can facilitate improvement of oral functions, depending on how they are used and maintained. Although, denture use patterns in older adults with dementia was explored in only a few studies, an inconsistent pattern of denture use and an overall poor denture hygiene was reported. The study by Campos et al.^35^ found that people with dementia frequently wore dentures in both arches. However, Srisilapanan and Jai-Ua^27^ showed that despite presenting with least one missing tooth, less than half of participants wore dentures. In those who did wear a removable prosthesis, Campos et al.^35^ noted that the quality of the dentures used was very poor. In fact, Hatipogulu et al.^24^ reported that people with dementia were more likely to experience issues such as stomatitis, which was due to poor hygiene practices, including not removing dentures at night.

In addition, Chen et al.^41^ reported that people with cognitive impairment, including dementia, had more carious teeth and retained roots compared to those with normal cognitive function. Similar findings were also reported by Chu et al.^32^ who found that people with AD presented with more carious teeth compared to controls with normal cognition. Although most of the cases reported by Chu et al.^32^ had healthy oral mucosal status, their mean unstimulated salivary flow rate was significantly lower compared to controls. Campos et al.^42^ also reported that salivary flow rate was lower in the AD group than in the control group. Reduced salivary flow, dry mouth and use of xerostomic medications were shown to present a significant risk of deterioration of oral health as evidenced by Lexomboon et al.^36^ who found that the use of xerostomic medication, even up to 3 years prior to a dementia diagnosis, increased the need for tooth extraction. However, no significant associations between xerostomic medication use and the need for dental restorations or preventive procedures were found.

In terms of oral and dental health status of those with different types of dementia, Syrjala et al.^25^ showed that people with AD had more teeth than those with vascular dementia or other types of dementia. They also presented with poorer oral hygiene, as well as denture hygiene, compared to other dementia groups. However, the results do not allow clear estimations of how these parameters relate to community dwellers with different types of dementia as only half of the total participants recruited lived in community settings, with the rest in formal/institutional care.

In terms of decline in oral health in relation to dementia stage, Ribeiro et al.^26^ showed that those with advanced AD had more calculus and biofilm compared to people with milder dementia or no dementia. Interestingly, Srisilapanan et al.^27^ showed that dental caries was highest in the moderate dementia group, in comparison those in mild or severe disease stages. Although the authors attributed increased care support for those in severe dementia stage for the observed improvement in dental caries status, we could not find any evidence on level of care in relation to dementia decline and oral health outcome.

Studies exploring dental health of people with dementia across different residential settings in comparison to those living in the community, presented limited information on varied patterns of denture use and the number of missing teeth.^29^ In general, more nursing home residents, with one and eight teeth, wore removable partial dentures compared to community dwellers. However, the pattern was reversed in those with more teeth remaining, with more community dwellers reporting partial denture use compared with nursing home residents.^29^

Overall, evidence suggests that older adults with dementia have higher risk of dental caries, tooth loss and periodontal disease, which impacts their capacity to chew and consume food. Although a varied pattern of denture use to replace masticatory function was noted, the prosthesis were of poor quality, with a reported lack in denture hygiene practice. Further oral and dental health issues, such as, reduced salivary flow and the risk of dental extractions due to xerostomic medication use were also noted.

There was limited evidence to support the view that people with Alzheimer’s dementia had worse oral and dental health, compared to those with other dementia types. Also, a varied risk of oral and dental health deterioration with respect to the dementia severity and a difference in oral health behaviour in relation to residential settings were noted. Even though, more research is needed on difference in oral health risk in relation to dementia severity and residential arrangements, a generally poor oral and clinical dental health status was reported in the studies we reviewed.

### Quality assessment

Using the MMAT tool guidance, an overall quality score was given for each study. Although two studies reported using a mixed-method approach,^29,31^ neither provided information on qualitative methods used or data collected. Therefore, we assessed these papers as quantitative.

Only one,^38^ out of 19 papers met all of the five quality criteria scoring a 5* rating. The remaining studies were largely 3* ^24,27,32,35,36,39,41^ or 4* ^28,31,33,34,40,42^ with the remainder scoring 2* ^26,30,37^ or below.^25,29^ Details of the quality assessed using the MMAT criteria are presented in Table 3.

**Table 3:** Methodological quality criteria scoring using the MMAT (2018)^31^ tool

Author, date	Criteria from the mixed methods appraisal tool							Overall quality score
	Screening questions (all study type)		Quantitative non-randomised					
	S1. Are there clear research questions?	S2. Do the collected data allow to address the research questions?	3.1. Are the participants representative of the target population?	3.2. Are measurements appropriate regarding both the outcome and intervention (or exposure)?	3.3. Are there complete outcome data?	3.4. Are the confounders accounted for in the design and analysis?	3.5. During the study period, is the intervention administered (or exposure occurred) as intended?	Star/percentage (%) quality criteria met
Araújo, 2020^39^	1	1	1	1	1	0	0	***
Gao, 2020^38^	1	1	1	1	1	1	1	*****
Emanuel, 2018^37^	1	0	0	0	1	0	1	**
Lexomboon, 2018^36^	1	1	1	0	1	1	0	***
Campos, 2018^42^	1	1	1	1	1	0	1	****
Campos, 2017^40^	1	1	1	1	1	0	1	****
Campos, 2016^35^	1	1	0	1	1	0	1	***
Luo, 2015^33^	1	1	1	0	1	1	1	****
Lee, 2015^34^	1	1	1	1	1	0	1	****
Chu, 2015^32^	1	1	1	1	0	1	0	***
Chen, 2015^41^	1	1	1	1	0	0	1	***
Del Brutto, 2014^31^	1	1	1	0	1	1	1	****
Cicciù, 2013^30^	1	1	1	0	0	0	1	**
Chen, 2013^29^	1	0	0	0	1	0	0	*
Lee, 2013^28^	1	1	0	1	1	1	1	****
Srisilapanan, 2013^27^	1	0	0	1	1	0	1	***
Ribeiro et al, 2012^26^	1	1	0	0	1	0	1	**
Syrjala, 2012^25^	1	1	1	0	0	0	0	*
Hatipoglu, 2011^24^	1	1	0	1	1	0	1	***

## Key findings

The major findings presented across all included studies were explored in relation to our research questions about oral health-related practices, beliefs and views, experiences and changes over time of community-dwellers with dementia. Key findings are summarised in Table 2b.

### What do older adults with dementia and their carers do to look after their oral health?

Only seven studies^24,27,29,32,33,38,41^ provided information regarding oral health-related practices in those living with dementia. Information regarding tooth brushing habits, use and care of dental prostheses and care assistance required were obtained from the studies.

#### Daily oral care (with or without assistance)

Studies showed that older adults with dementia living in their own homes with community interactions presented with poor oral hygiene practice—with reported difficulties in their ability to self-perform daily oral care and so they needed care-assistance. Dementia-related cognitive and functional issues including forgetfulness, unwillingness to brush, as well as lack of dexterity were identified as the difficulties people with dementia faced in performing daily oral hygiene practice.^32^

Similarly, Gao et al.^38^ found that only 3% of the participants with dementia brushed their teeth daily due to difficulties with tooth brushing. In comparison, 100% of controls without dementia stated that they brushed their teeth every day. Also, the number of people requiring care assistance was higher in the dementia group compared to controls. Although, Srisilapanan and Jai-Ua^27^ reported that a majority of people with dementia were functionally independent, with normal hand and arm functions, only half of them were able to perform oral care by themselves. The evidence was further supported by Chen et al.^41^ who found that community dwellers with cognitive impairment including dementia had a greater need for carer assistance to maintain their oral hygiene, which was directly related to their risk of dental caries or retained roots. In fact, the loss of ability to self-perform oral hygiene significantly increased the risk of poor oral health in these groups, reinforces the need for improved carer support.

Similar findings were also reported in an earlier study by Chen et al.^29^ when comparing people with dementia living in different care settings. Even though community dwellers demonstrated better capacity for oral hygiene practice compared to those in assisted living or nursing homes, participants living in community required supervision, while some were non-cooperative to carer support. In one population-based study by Luo et al.^33^ it was found that older adults with dementia scored significantly higher on Activity of Daily Living Scale (ADL) compared to people without dementia or those with mild cognitive impartment, indicating a greater need for care and assistance for the dementia group. In fact, Hatipoglu et al.^24^ showed that low cognitive function was more strongly related to oral and dental practices than reduced functional assessment scores.

Therefore, regardless of their functional independence or residential arrangements, people with dementia had poor oral hygiene practices and relied on caregiver assistance to look after their oral and dental health. The loss in cognitive function due to dementia was found to be a more significant predictor of poor oral health practice than their functional abilities.

### What are their beliefs and views about oral health/oral health care?

Although eight studies^26,28,30,31,34,35,37,39^ provided information on the views and beliefs of those with dementia, they were mostly focused on understanding their self-perceived quality of life in relation to their oral and dental status. However, there was a lack of evidence on their perception about the importance of oral health care and areas of care needs.

#### Self-perceived oral health and oral health-related quality of life

Across the five studies^26,28,30,35,39^ that assessed self-perceived Oral Health Related Quality of Life (OHRQoL) in community residents with dementia, the evidence varied. Lee et al.^28^ found that people with mild dementia viewed and reported their quality of life as significantly lower than participants with normal cognitive function, even after controlling for confounders. Ciccu et al.^30^ identified issues such as difficulty in speaking, self-consciousness due to oral health problems, concerns about their oral status and limiting contact with people because of the condition of oral health were the most significant factors which influenced how people with dementia viewed their quality of life. In addition, clinical parameters of periodontal disease destruction, such as gingival bleeding and increased probing depth, were also identified as factors that negatively impacted those with dementia, who reported as being dissatisfied about their oral health.^30^ Furthermore, Lee et al.^28^ found that in people with mild dementia, higher GOHAI scores representing better self-perceived oral health, were correlated with better clinical disease status. In particular, the total number of decayed root surfaces was the most significant correlate of the participants’ oral health perception, with those with more decayed coronal surfaces reporting lower GOHAI total scores. Therefore, we found that when asked to subjectively evaluate their health and wellbeing, people with dementia reported a number of clinical, functional and aesthetic factors related to their oral health, which negatively influenced their quality of life.

In contrast, in four, albeit smaller studies, there was evidence that people with dementia had better perception about their oral health, and oral health-related quality of life.^26,35,39,42^ Ribeiro et al.^26^ found that people in severe stages of dementia scored higher on GOHAI index, indicating that they had a more positive view of their oral health. Although, this group also viewed their quality of life as similar to those without dementia, they were more reliant on their caregiver support and required assistance in answering questions. Similarly, Araujo et al.^39^ found that despite having fewer teeth and more severe periodontitis, people with mild or moderate AD reported more positive GOHAI scores when compared with their family members without dementia. Furthermore, Campos et al.^42^ found that when measuring masticatory functions, people with AD consistently reported higher GOHAI scores despite a compromised masticatory efficiency. These findings raise important issues about the capacity of older with dementia to correctly identify and communicate their needs. The fact that despite poor oral health and dental disease status, people with dementia seemed less aware of these issues, raises concerns about missed opportunities to address undetected and often preventable oral health problems.

Interestingly, Campos et al.^35^ noted that caregivers also had similar views about the quality of life of those they were caring for, and they too reported it as more favourable than clinically verified. Although, the total GOHAI score and score for psychological domain showed high inter-rater agreement between patient and caregiver, domains such as speaking, biting and food consumption difficulties were less strongly correlated. Therefore, the caregiver group was also less aware of the range of oral health issues present.

#### Self-rated appearance in relation to their oral health

Only one study reported on the views of people with dementia about their oral health appearance.^39^ It showed that people in early stages of dementia generally viewed their appearance as good, although this changed as their dementia progressed. However, information about how this compares to the control group, which included their family members, was not available.^39^

#### Self-perceived social support

Lee et al.^34^ highlighted that people with dementia perceived their social network as weaker and they also had lower confidence in their social network compared to those without dementia.

### What are their oral health-related experiences?

#### Experience of accessing professional care

Only two studies provided an insight into the experience of older adults diagnosed with dementia in terms of accessing professional care. An American based study by Lee et al.^34^ reported that nearly half of all participants with dementia had no regular schedule of attending a dentist, which was lower compared to of those without dementia. This was also affected by the fact that most people with dementia did not have dental insurance to cover the treatment costs. In contrast, a study conducted in England by Emanuel and Sorensen^37^ found that despite a dementia diagnosis, older adults viewed their oral health as important and were keen on continuing their professional care. In fact, most of participants with dementia regularly attended a dental practice and about half of all patients attended for regular hygienist sessions. However, those who did attend professional services did not always receive routine preventative care and advise including fluoride supplement and dietary or oral hygiene advice. It is worth noting that the two studies that reported on professional care attendance were conducted in England and the USA, which have different healthcare systems and social support provisions. Although we are unable to draw direct comparisons or robust conclusions due to small sample size and heterogeneity, further research in the field is required to understand how care attendance could be implicated by social and financial support for those requiring care. Nevertheless, the evidence from the study in the UK shows a clear scope to improve prevention and care for people with dementia, even in countries with more facilitative social care systems.

### Changes over time

Only one study^36^ reported on changes over time and did so in relation xerostomic medication and dental health. Although, the change was assessed in relation to progression of dementia, they found that use of xerostomic medication used prior to a diagnosis of dementia (up to 3 years) resulted in poorer oral health in a longitudinal dose-response manner.

## Discussion

One of the most notable findings of this rapid review is the lack of robust research evidence on the oral health-related views/beliefs, self-care or assisted care practices, as well as experiences of professional care of community dwellers with dementia. This paucity of evidence was reflected in our screening process, whereby only 19 out of the 57 full texts reviewed met our criteria. The main reasons for exclusion were lack of clarity regarding inclusion of participants with dementia, with studies recording a dementia diagnosis, but then excluding the participants from data collection. These issues are not unique to our study as highlighted by earlier systematic reviews, which found that people with cognitive decline and dementia are frequently excluded from research, due to often poorly justified concerns surrounding safeguarding issues and ethical challenges.^45,46^ A move towards more flexible research approach and adaptive study consent strategies have made way for greater research inclusivity,^47^ which also need to be reflected in practice. Also, as most of the studies we reviewed were cross sectional or case-control, we note a lack of longitudinal data on oral health change in relation to dementia progression. Observational studies that are cross-sectional or case-control in design allow collection of data at a single point in time as a snapshot overview. However, it does not provide information about changes over time to be captured, as allowed by a longitudinal study design. This may be an important consideration for studies involving those with neurodegenerative disorders including dementia, which can rapidly progress within a short timeframe. Moreover, normative data collected through quantitative methods need to be supplemented by in depth exploration of the perspectives of those with dementia, to support an overall picture of their oral care needs and explore any dissonance with reported health-related quality of life. In this regard, we recommend future studies to consider using a longitudinal, mixed research methods to fully capture the psycho-social aspects of oral health care and changes over time for those with a clinical diagnosis of dementia. Studies should also consider including the views and experiences of their carers, both formal and informal.

Although most of the studies we reviewed were clinically focused, they did however confirm that community dwellers with dementia generally have poor professionally assessed oral health (notably missing teeth, dental caries and periodontal disease risk). To maintain good oral and dental health, daily tooth brushing, limiting sugary diet, using fluoride supplements, as well as, removal and cleaning of dentures are essential.^48,49^ Our findings on oral health practices, where available, showed that most people with dementia do not brush their teeth daily and they are less able to look after their dentures due to cognitive and functional challenges. The loss of cognitive function, even in mild stages of dementia, may present issues with language, memory, attention, and apraxia, which hinders their ability to self-perform oral care.^50^ In addition, physical impairments, including a gradual decline in manual dexterity and motor skills also impacts their ability to perform oral and personal self-care.^51^ A generally poor clinical disease status (which we noted, regardless of dementia type, severity and community residential setting) is an important indication of substandard oral care practices and a reduced capacity for self-performed care. This is unsurprising, given our findings, which suggested that even those who were functionally independent required caregiver supervision. Therefore, a major influence on the oral health practices of those with dementia is shaped by their reliance on their caregivers, who may not have the necessary skills, knowledge and training to provide appropriate oral health care.^52,53^ However, the lack of information regarding the level and type of caregiver support that is available to these community dwellers limits our overall understanding of their daily self-care and assisted care practices to maintain their oral health.

What people with dementia and their carers do to take care of their mouth can inadvertently be affected by how they view their oral health and perceive its impact on their quality of life. The conflicting findings in self-reported oral health and clinically verified dental status, across the different studies we reviewed, raise important concerns about the ability of people with dementia to recognise and communicate their oral care needs. Issues such as progressive memory loss and personality changes are unique to those with dementia and can have an effect on how they view their health and wellbeing.^54^ Furthermore, as dementia progresses, individual preferences and perspectives can be harder to determine due additional communication and behavioural barriers.^55^ Therefore, it is important to raise awareness of the importance of oral health care and provide routine professional care for people living with dementia. It is equally important to ensure that their primary caregiver or family members are adequately supported to identify early signs of problems and arrange suitable clinical care.

As a proactive approach, integrating dialogues about oral health care at the earliest signs of cognitive decline means that effective care pathways can be established while people are able to partake in making decisions about their own health and health care. Effective intervention plans will also require training carers with a goal of promoting and maintaining health; not just the oral health of vulnerable groups, but also the overall general health and wellbeing of those people for whom they care.^56,57^ This may be significant for those living in their own homes and communities while being looked after by informal caregivers, mostly spouses and children, who need additional professional support and advice on how to direct timely care across dental care pathways.^57^

Access to dental services is an important concern as dental professionals are uniquely placed to create, and maintain long-term relations with the patients they care for, providing opportunities to have meaningful discussions about the dental care provisions for later disease stages.^55^ Dental care professionals also have an important role of engaging and instructing carers alongside those with dementia to implement effective oral care routine in consideration of patient wishes and care needs.^48^ It is therefore important to understand how community dwellers and their carers engage with professional services and what services are made available to them. Although this rapid review highlights a lack of emphasis on professional care access, one study did provide evidence that where dental services were offered as a part of routine geriatric care, patients’ oral health status was better than the national profile.^27^ Recent systematic reviews have also reinforced the need for timely professional care using both subjective and objective measures of health assessment, in order to capture the range of an age-related loss of oral health function due to cognitive and functional decline in people with dementia.^58,59^

Furthermore, we also note an equity dimension to care access influenced by the socio-economic structures and payment models for those with dementia, which could translate into care access behaviour. Although, there are methodological and statistical differences across studies we reviewed, which do not allow a direct comparison, these findings do raise important questions about health system issues and socio-economic barriers affecting professional care access. This resonates with a recent research paper from our research group which highlights similar challenges and reinforces the need for efficient integrated systems that provide a clear pathway for people with dementia to access and navigate care services.^60^ Therefore, useful signposts to dementia friendly dental services, if available, would be of significant advantage to improve access to professional support. Also, existing health care systems should be reviewed to provide necessary training and support for the dental workforce who are challenged with the critical task of supporting the health of the most vulnerable people in society.^60^ Further research and action in this field are urgently required.

## Strengths and limitations

This review has several strengths, which should be acknowledged. First, the review process was conducted by a multidisciplinary team containing researchers, dental and care professionals, and public health expertise. Second, to the best of our knowledge, this is a first review in a high priority area with emerging research evidence. Therefore, the review provides helpful directions and suggestions for future studies in the field.

We also acknowledge the limitation of our search strategy that resulted in capturing a large volume of studies. The strategy was refined in consultation with the library services to ensure that the minimum number of papers was identified whilst gaining maximum coverage to address the research questions. Furthermore, whilst it could be argued that the restriction to the 10-year period may have excluded any earlier papers on the subject, the backwards/forwards search did not reveal any further early data for the review. In addition, inclusion of three papers from the same author group and two from the same study may have introduced bias, which may implicate the generalisability of findings.

## Conclusion

Although there was evidence on the oral health care needs of those living with dementia in their communities, there was limited evidence on their oral health-related practices, beliefs/views and experiences. The available evidence suggests that the oral and dental health of this population is generally poor and their ability for self-care (notably oral hygiene) reduces with cognitive decline. With regards to self-perceived oral health and quality of life, the evidence varied, with noted discrepancies in relation to their normative care needs. Few studies also reported a low self-perception in people with dementia, who also viewed their social support as weak. Research evidence, albeit weak, suggests a reduced capacity of people with dementia and their carers to correctly identify and report oral health-related issues experienced by this population. There was a paucity of evidence on dental care experience with dental access appearing system dependent. Only one study investigated changes over time and highlighted an increased risk of oral health deterioration in relation to the use of xerostomic medication use.

Overall, the limited evidence on perspectives of a largely underrepresented population group with clear oral health care needs highlights an urgent need for more methodologically robust research in the field. Further studies should consider mixed-method approaches, to capture multiple perspectives of people with dementia and to bring their voice to the forefront. Also, longitudinal studies would be useful to capture the unique lived experiences of people living with dementia and identify how they change over time.
