# Correction: Perspectives of community-dwelling older adults with dementia and their carers regarding their oral health practices and care: rapid review

**DOI:** 10.1038/s41405-021-00092-3

**Published:** 2021-12-14

**Authors:** S. KC, M. Aulakh, S. Curtis, S. Scambler, J. E. Gallagher

**Affiliations:** Faculty of Dentistry, Oral & Craniofacial Sciences, King’s College London, Denmark Hill Campus, Bessemer Road, London, SE5 9RS UK

**Keywords:** Dentistry, Gerodontics

Correction to: *BDJ Open* 10.1038/s41405-021-00091-4, published online 22 November 2021.

When originally published on 22 November 2021, there were errors in the following sections:

‘Introduction’ section: “Also, the number of ‘edentate’, adults with no natural teeth, had fallen to 6%, which represents an all-time recorded low” should have read “Also, the volume of ‘edentate’ adults had fallen to 6%, which represents an all-time recorded low”.

‘Oral and dental disease’ section: “Although, most of the cases reported by Chen^32^ had healthy oral mucosal status, their mean unstimulated salivary flow rate was significantly lower compared to controls” should have read “Although most of the cases reported by Chu et al.^32^ had healthy oral mucosal status, their mean unstimulated salivary flow rate was significantly lower compared to controls”.

‘Quality assessment’ section: “Although two studies reported using a mixed-method approach, neither provided information on quantitative methods used or data collected. Therefore, we assessed these papers as quantitative” should have read “Although two studies reported using a mixed-method approach, neither provided information on qualitative methods used or data collected. Therefore, we assessed these papers as quantitative”.

‘What are their oral health-related experiences?’ section: “Nevertheless, the evidence from the study in the UK shows a clear scope to improve oral health prevention and care for people with dementia, even in countries with more facilitative social care systems” should have read “Nevertheless, the evidence from the UK shows a clear scope to improve prevention and care for people with dementia, even in countries with more facilitative social care systems”.

‘Discussion’ section: “In addition, physical impairments, including a gradual decline in manual dexterity and motor skills also implicates their ability to perform oral and personal self-care” should have read “In addition, physical impairments, including a gradual decline in manual dexterity and motor skills also impacts their ability to perform oral and personal self-care”. Also, “Effective intervention plans will also require training carers with a goal of promoting not just the oral health of vulnerable groups, but also the overall general health and wellbeing of those that they care for” should have read “Effective intervention plans will also require training carers with the goal of promoting and maintaining health; not just the oral health of vulnerable groups, but also the overall general health and wellbeing of those people for whom they care”.

‘Conclusion’ section: “Also, longitudinal studies may be useful to capture the unique lived experiences of people living with dementia and identify how it changes over time” should have read “Also, longitudinal studies would be useful to capture the unique lived experiences of people living with dementia and identify how they change over time”.

The authors apologise for any inconvenience caused.

